# Predictors of survival in pediatric intensive care unit in a low-income country: A retrospective cohort study

**DOI:** 10.1371/journal.pone.0315863

**Published:** 2025-02-12

**Authors:** Mohamedawel Mohamedniguss Ebrahim, Abdikarin Ahmed Mohamed, Mohammed Mustefa Mohammedamin

**Affiliations:** 1 School of Medicine, College of Health Sciences, Mekelle University, Mekelle, Ethiopia; 2 Department of Pediatrics and Child Health, Mogadishu Somalia Turkish Training and Research Hospital, Mogadishu, Somalia; University of Sao Paulo: Universidade de Sao Paulo, BRAZIL

## Abstract

**Background:**

The goal of a pediatric intensive care unit is to treat life-threatening illnesses. Yet, there is a lack of data on survival rates and factors influencing survival in pediatric intensive care units (PICUs) in low-income countries like Ethiopia.

**Objective:**

The purpose of this study was to evaluate survival and its predictors in the pediatric intensive care unit of Ayder Comprehensive Specialized Hospital, Tigray, Ethiopia.

**Method:**

A retrospective cohort study was implemented on a total of 223 patients admitted to the PICU from September 2019 to August 2020. Using a checklist, trained healthcare workers gathered secondary data from patient charts. The dependent variable was time-to-death. EpiData 4.6 and STATA 16 were used for data entry and data analysis, respectively. Descriptive statistics, cumulative incidence, incidence density, median survival time, and adjusted hazard ratio were calculated to describe variables, estimate mortality rate and risk, and identify factors associated with survival. P<0.05 was used to declare a statistically significant relationship.

**Result:**

A total of 46 (20.6%) deaths were recorded in this study. The cumulative incidence of death was higher in patients transferred from the ward to the intensive care unit (34.7%). The death rate was higher in patients with cardiac problems (40.0%), followed by oncological diseases (35.0%) and infectious diseases (27.9%). The adjusted risk of death was 3.3 times higher in pediatric patients with heart problems than in patients without heart problems. Again, the adjusted risk of death in children with a Glasgow Coma Scale (GCS) of 9 to 14 and a GCS below 9 was 2.2 and 2.5 times higher, respectively, than in children with a GCS of 15. Finally, children with endotracheal tubes were about five times more likely to die than children without endotracheal tubes.

**Conclusion:**

It’s critical to diagnose and treat cardiac issues in pediatric patients as early as possible. Patients with low GCS scores require careful observation. Alternative strategies should be taken into consideration in order to lessen the need for endotracheal tubes and enhance results.

## Introduction

The pediatric intensive care unit (PICU) is used to treat life-threatening illnesses. In 1955, Dr. Goran Haglund opened the first intensive care unit for a child who had to be operated on because of a ruptured appendix. The patient fell into a coma postoperatively and was treated for shock and respiratory failure. Since then, the intensive care unit has become a standalone area with specialized equipment and medical professionals to save people in need of advanced medical and surgical care [1,2].

Although child mortality rates worldwide have declined significantly, from 93 per 1,000 live births in 1990 to 39 in 2018, sub-Saharan Africa still struggles with high mortality rates. One reason for this disparity could be the financial costs of intensive care. Per capita health expenditure in low- and middle-income countries (LMIC) is US$90 compared to US$5266 in high-income countries (HIC) [3–5].

A study conducted in a neighboring country to assess the outcomes of pediatric patients in the intensive care unit found a high mortality rate of 37.9%. Of the survivors, 18.9% developed a new disability upon discharge from the intensive care unit [6]. Studies in Ethiopia assessing the survival status of the pediatric ICU population reported a mortality rate of 28.6%, 32.6%, and 43.8%. Intensive care unit outcomes were determined by clinical status at admission, patient age, and comorbidities, as well as factors reported during ICU admission such as: the use of mechanical ventilation, the need for inotropic support, the level of consciousness, the length of stay in the intensive care unit, and complications during the stay in the intensive care unit, such as circulatory and ventilation-related respiratory complications [7–9].

The paucity of published data on pediatric critical care in low- and middle-income countries (LMICs), particularly Ethiopia, makes it difficult to alter practice and enhance results. Therefore, this study’s goal was to look into factors that are predictive of survival in pediatric intensive care units.

## Method and materials

### Study area, study design, and study period

A retrospective cohort study was conducted in the pediatric intensive care unit (PICU) of Ayder Comprehensive Specialized Hospital (ACSH) in Tigray Regional State, northern Ethiopia. The entire population of Tigray, as well as residents of other regions such as Amhara Regional State, Afar Regional State, and Eritrea, are served by this hospital. The study period extended from September 2019 to August 2020.

### Study population

This study included and examined 223 pediatric patients who were hospitalized in the pediatric intensive care unit during the study period and had complete medical records. The medical records were accessed from June 1, 2021 to August 31, 2021.

### Study variables

The dependent variable was time to death. Time was measured in days and calculated as the difference between the discharge or death date and the admission date. Explanatory variables were sociodemographic characteristics, comorbidity, admission diagnosis, nutritional status, Glasgow Coma Scale (GCS), patient feeding method, procedure (use of a mechanical ventilator, urinary catheter, central catheter, chest tube, etc.), use of antibiotics, hospital-acquired infection (HAI), and length of hospital stay.

### Data collection, management and analysis

Data were collected by trained health workers using a checklist. The data source was electronic medical records and patient files. EpiData 4.6 was used for data entry. We used STATA 16 for data cleaning and analysis. The cumulative incidence of death was calculated for different categories of patients. The difference in survival between groups was evaluated statistically and graphically using the log-rank test and Kaplan-Meir curves. The death rate was calculated per 100 patient days. The length of hospital stays between those who died and those discharged alive was described using the median and interquartile range (IQR).

The relationship between predictors and time to death in the pediatric intensive care unit was analyzed using a multivariate Cox proportional hazard regression model. Adjusted hazard ratios (AHR) with 95% confidence intervals (CI) and p values were used to measure the association between time to death in the pediatric intensive care unit and predictors. For variables with p<0.05, a statistically significant relationship was declared. Schoenfeld residuals and Martingale residuals were used to evaluate the proportional hazards assumption and model fitness. A variance inflation factor (VIF) value less than 5 was used to determine the absence of multicollinearity.

### Ethical clearance

Ethical approval was obtained from the Institutional Review Board (IRB) of the College of Health Sciences at Mekelle University with IRB number ERC:1853/2021. A letter of approval was also obtained from the ACSH Chief Clinical Director’s office before the commencement of the study. Caregivers provided informed verbal consent after receiving a detailed explanation of the study’s objectives and purpose. They were also informed of their right to withdraw participation at any time. To protect participant privacy, personal identifiers were replaced with codes during the de-identification process. Collected data remained confidential and securely stored on password-protected computers, with no unauthorized third-party access.

## Result

**Fig 1:**
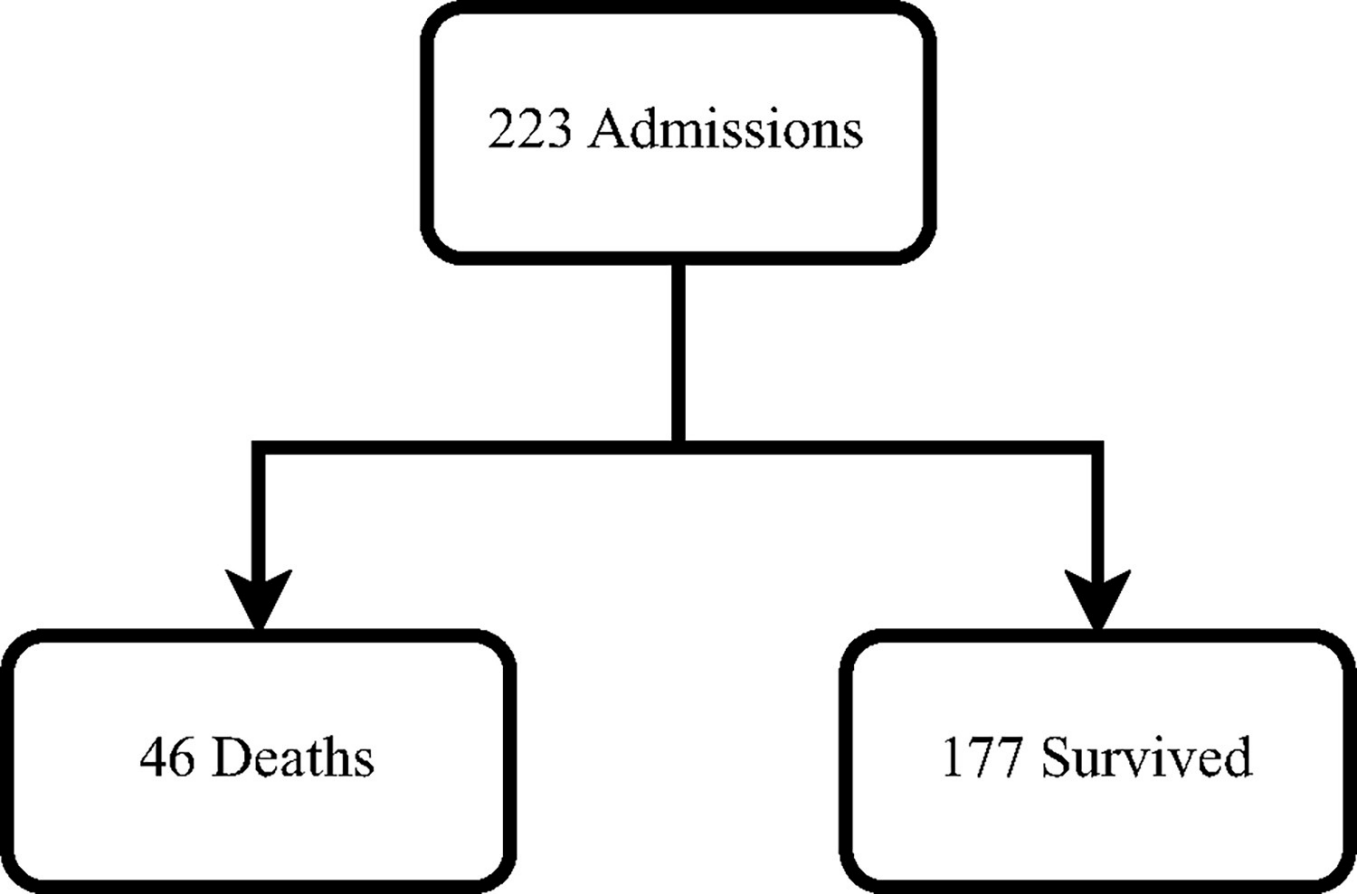
Flow chart of deaths and survivals in PICU of ACSH, N = 223.

A total of 223 pediatric patients admitted to the intensive care unit of Ayder Comprehensive Specialized Hospital (ACSH) were analyzed for this study. A total of 46 (20.6%) deaths were recorded (Fig 1).

### Sociodemographic characteristics

In this study, 138 (61.9%) of the patients were male. The risk of death was 27.1% in women and 16.7% in men. However, the probability of survival did not differ significantly between men and women (log-rank test: p = 0.282). Again, there was no statistically significant difference in survival probability between age groups. Patients from rural areas accounted for 63.7% of pediatric intensive care unit admissions. Most admissions came from emergency room 151 (67.7%), followed by ward 49 (22.0%). The cumulative incidence of death was higher in patients transferred from the ward to the intensive care unit (34.7%), but the difference in survival probability between sources was not significant (log-rank test: p = 0.064) (**Table 1**).

**Table 1:** Sociodemographic-related characteristics in relation to cumulative incidence of death and survival probability of patients admitted to the pediatric ICU of ACSH, N = 223.

*Characteristics*		*Alive*	*Dead*	*Total*	*Log-rank test*
Gender					0.282
	Female	62 (72.9)	23 (27.1)	85	
	Male	115 (83.3)	23 (16.7)	138	
Age category					0.733
	<1 Year	52 (82.5)	11 (17.5)	63	
	1–4 Years	49 (79.0)	13 (21.0)	62	
	5–9 Years	41 (78.8)	11 (21.2)	52	
	10–17 Years	35 (76.1)	11 (23.9)	46	
Residence					0.485
	Rural	111 (78.2)	31 (21.8)	142	
	Urban	66 (81.5)	15 (18.5)	81	
Source of admission					0.064
	Emergency	122 (80.8)	29 (19.2)	151	
	Ward	32 (65.3)	17 (34.7)	49	
	Operation theatre	23 (100.0)	0 (0.0)	23	

### Organ system affected and Glasgow coma scale

The most common reasons for admission to the pediatric intensive care unit were central nervous system-related problems 57 (25.6%), followed by infectious diseases 43 (19.3%), and respiratory problems 30 (13.5%). However, the cumulative incidence of death was higher in patients with cardiac problems (40.0%), followed by oncological diseases (35.0%) and infectious diseases (27.9%). The probability of survival also differed statistically significantly between the affected organs/systems (log-rank test: p = 0.039). The cumulative incidence of death was about 21% higher in patients with heart problems (log-rank test: p = 0.006). The risk of death was significantly higher in patients with a lower Glasgow Coma Scale (log-rank test: p = 0.002). The risk of death in patients with comorbidities was 32 (25.2%) (**Table 2**).

**Table 2:** Organ/System-affected related characteristics in relation to cumulative incidence of death and survival probability of patients admitted to the pediatric ICU of ACSH, N = 223.

*Characteristics*		*Alive*	*Dead*	*Total*	*Log-rank test*
Organ/System affected					0.039
	CNS	47 (82.5)	10 (17.5)	57	
	Respiratory	27 (90.0)	3 (10.0)	30	
	Cardiac	12 (60.0)	8 (40.0)	20	
	Renal	11 (73.3)	4 (26.7)	15	
	Gastro-intestinal	18 (90.0)	2 (10.0)	20	
	Infectious	31 (72.1)	12 (27.9)	43	
	Oncologic	13 (65.0)	7 (35.0)	20	
	Other	18 (100.0)	0 (0.0)	18	
Cardiac problem					0.006
	Yes	12 (60.0)	8 (40.0	20	
	No	165 (81.3)	38 (18.7)	203	
GCS					0.002
	15	98 (87.5)	14 (12.5)	112	
	9–14	50 (71.4)	20 (28.6)	70	
	<9	21 (65.6)	11 (34.4)	32	
	Sedated	8 (88.9)	1 (11.1)	9	
Nutritional status					0.076
	Normal	99 (86.8)	15 (13.2)	114	
	Moderate acute malnutrition	32 (74.4)	11 (25.6)	43	
	Severe acute malnutrition	40 (70.2)	17 (29.8)	57	
	Not recorded	6 (66.7)	3 (33.3)	9	
Comorbidity					0.193
	Yes	95 (74.8)	32 (25.2)	127	
	No	82 (85.4)	14 (14.6)	96	

### Management

The number of patients who received maintenance fluids were 98 (44.0%), and the cumulative incidence was higher in this patient group (26.5%) than in patients who were fed orally (6.3%) or through a nasogastric tube (22.1%) (log-rank test: p = 0.017). There were 42 (18.8%) patients who were intubated (via endotracheal tube), and in this group, up to 60% died. The probability of survival was extremely lower in this patient group (log-rank test: p < .001). Upon admission, antibiotics were started immediately in 190 (85.2%) patients (**Table 3**).

**Table 3:** Management-related characteristics in relation to cumulative incidence of death and survival probability of patients admitted to the pediatric ICU of ACSH, N = 223.

*Characteristics*		*Alive*	*Dead*	*Total*	*Log-rank test*
Patient feeding					0.017
	Oral feeding	45 (93.8)	3 (6.3)	48	
	Nasogastric feeding	60 (77.9)	17 (22.1)	77	
	Maintenance fluid	72 (73.5)	26 (26.5)	98	
Mechanical ventilator					<0.001
	Yes	17 (42.5)	23 (57.5)	40	
	No	160 (87.4)	23 (12.6)	183	
Urinary catheter					0.089
	Yes	19 (61.3)	12 (38.7)	31	
	No	158 (82.3)	34 (17.7)	192	
Central line catheters					0.548
	Yes	2 (66.7)	1 (33.3)	3	
	No	175 (79.5)	45 (20.5)	220	
NG tube					0.439
	Yes	99 (78.0)	28 (22.0)	127	
	No	78 (81.3)	18 (18.8)	96	
Endotracheal tube					<0.001
	Yes	17 (40.5)	25 (59.5)	42	
	No	160 (88.4)	21 (11.6)	181	
Chest tube					0.275
	Yes	7 (87.5)	1 (12.5)	8	
	No	170 (79.1)	45 (20.9)	215	
Antibiotics on admission					0.316
	Yes	147 (77.4)	43 (22.6)	190	
	No	30 (90.9)	3 (9.1)	33	

### Complications and length of hospital stay

The total number of patients who developed a hospital-acquired infection (HAI) was 45 (20.2%). The probability of survival was not significantly different between patients with HAI and patients without HAI (log-rank test: p = 0.739), although there was a 7% difference in the risk of death. The most common sites of infection in HAI were the lung and chest. Fever was reported in 17 (37.8%) patients with HAI. And, 37.8% of HAIs were detected by culture. The median length of hospital stay was 5.5 days for patients who died and twice (12 days) for patients discharged alive (**Table 4**).

**Table 4:** Complication-related characteristics in relation to cumulative incidence of death and survival probability of patients admitted to the pediatric ICU of ACSH, N = 223.

*Characteristics*		*Alive*	*Dead*	*Total*	*Log-rank test*
HAI					
	Yes	33 (73.3)	12 (26.7)	45	0.739
	No	144 (80.9)	34 (19.1)	178	
Culture-proven HAI					0.918
	Yes	12 (70.6)	5 (29.4)	17	
	No	21 (75.0)	7 (25.0)	28	
Focus of infection					
	Chest	17 (70.8)	7 (29.2)	24	0.306
	Urinary tract	2 (66.7)	1 (33.3)	3	
	GI	4 (100.0)	0 (0.0)	4	
	Other	10 (71.4)	4 (28.6)	14	
Symptoms of patients with HAI					
	Fever	15 (88.2)	2 (11.8)	17	<0.001
	Cough	4 (100.0)	0 (0.0)	4	
	Urinary compliant	0 (0.0)	2 (100.0)	2	
	Vomiting	1 (100.0)	0 (0.0)	1	
Hospital stays, median (IQR)		12 (13)	5.5 (11)	10 (14)	

### Predictors of survival in the pediatric ICU

**Fig 2:**
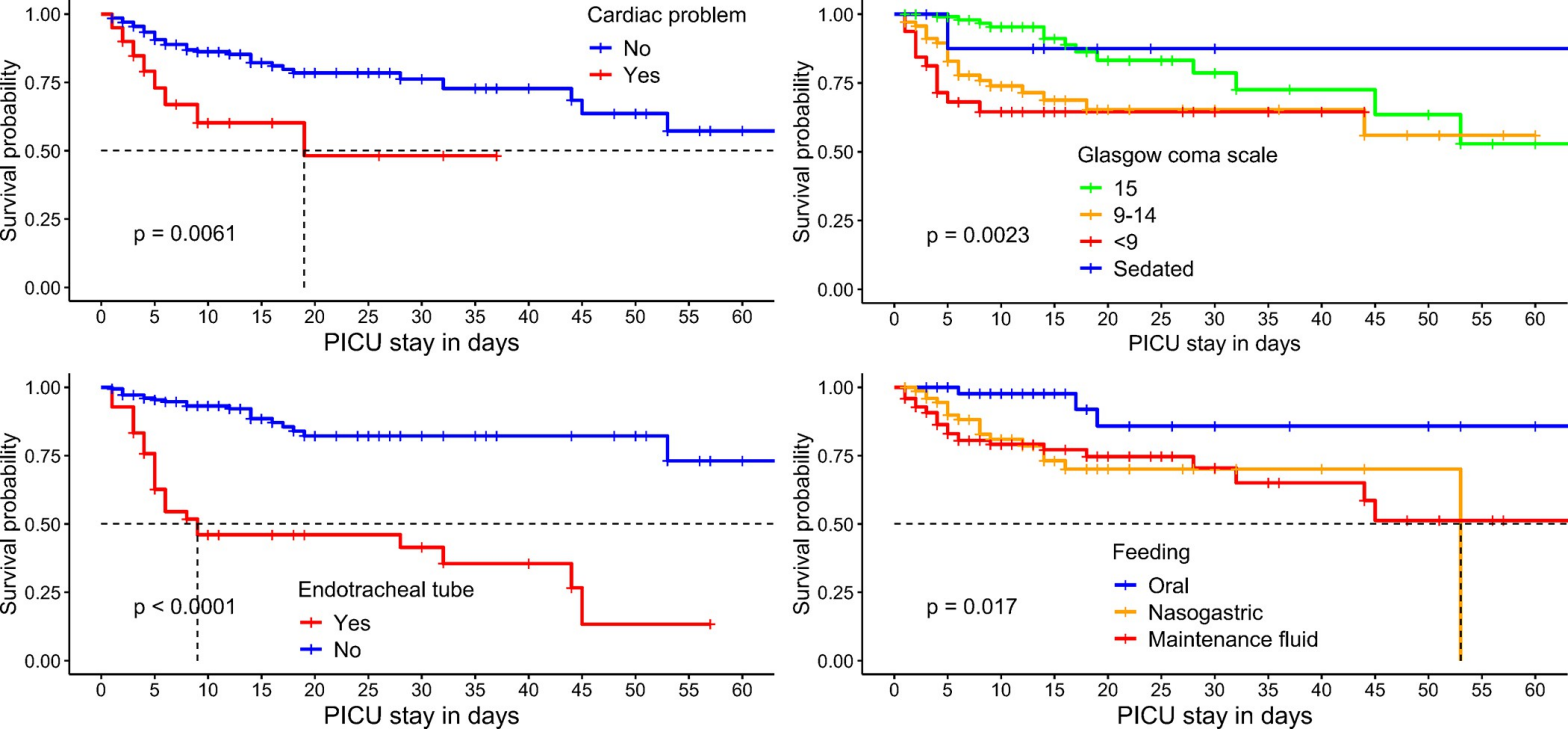
Kaplan-Meier survival curves for feeding method, use of endotracheal tube, glasgow coma scale, and presence of cardiac problem.

A multivariate Cox proportional hazards regression was fitted to identify significant predictors of time to death in the pediatric intensive care unit. The adjusted hazard of death was 3.3 times higher in pediatric patients with heart problems than in patients without heart problems. On average, the mortality rate for children with heart problems was three per 100 patient-days (**Table 5**). The median survival time for children with heart problems was 19 days (Fig 2).

**Table 5:** Predictors of time to death in patients admitted to the pediatric ICU of ACSH, N = 223.

*Predictors of* *time-to-death*		*Death rate per 100* *patient-days [95% CI]*	*Adjusted hazard ratio* *[95% CI]*	*p value*
Cardiac involvement				
	Yes	3.3 [1.7, 6.7]	3.3 [1.4, 8.2]	0.009
	No	1.1 [0.8, 1.6]	1	
Glasgow coma scale				
	15	0.7 [0.4, 1.3]	1	
	9–14	2.0 [1.3, 3.1]	2.2 [1.0, 4.6]	0.041
	<9	2.5 [1.4, 4.6]	2.5 [1.0, 6.2]	0.044
	Sedated	0.4 [0.1, 3.1]	0.9 [0.1, 7.5]	0.939
Comorbidity				
	Yes	1.5 [1.0, 2.1]	1.9 [1.0, 3.6]	0.059
	No	1.0 [0.6, 1.7]	1	
Patient feeding				
	Oral	0.3 [0.1, 1.0]	1	
	Nasogastric tube	1.7 [1.1, 2.7]	1.8 [0.5, 7.1]	0.373
	Maintenance fluid	1.6 [1.1, 2.3]	2.0 [0.5, 7.8]	0.292
Endotracheal tube				
	Yes	4.3 [2.9, 6.3]	4.6 [2.4, 8.8]	<0.001
	No	0.7 [0.5, 1.1]	1	

Likewise, the adjusted hazard of death in children with a Glasgow Coma Scale (GCS) of 9–14 and a GCS below 9 was 2.2 and 2.5 times higher, respectively, than in children with a GCS of 15. The last, but not the least significant predictor of time to death in the pediatric intensive care unit was intubation with an endotracheal tube. Children with an endotracheal tube had a risk of death about five times higher than children without an endotracheal tube (**Table 5**). The median survival time for children with an endotracheal tube was 9 days (Fig 2).

The goodness of fit test using martingale residuals is insignificant (*χ*^*2*^ (1) = 0.040, p-value = 0.842), meaning the multivariate model fits the data well. Both global and individual tests of the proportional hazard assumption tests were insignificant, meaning that the model satisfies the proportional hazard assumption (global Schoenfeld test: *χ*^*2*^ (8) = 5.63, *p-value* = 0.689). The multivariate model does not have a multicollinearity problem (maximum *VIF* = 2.23, mean *VIF* = 1.43).

## Discussion

The assessment of survival predictors in pediatric intensive care settings has not been well studied in sub-Saharan African countries, and the studies reported internationally on intensive care are not very numerous [10]. Because there is a lack of published data on pediatric critical care, it is difficult to improve practices and outcomes in LMICs like Ethiopia. The aim of this study is to examine predictors of survival in the pediatric intensive care unit of Ayder Comprehensive Specialized Hospital (ACSH), one of the most prestigious hospitals in Ethiopia’s Tigray regional state, serving nearly 3 million children under the age of eighteen.

It is estimated that 5.3 million children died worldwide in 2018. Furthermore, 80% of these deaths were reported in sub-Saharan Africa and Asia. Three of the five countries where half of global pediatric deaths occurred were countries in sub-Saharan Africa, increasing mortality in the region from 31% in 1990 to 54% in 2018 [10]. The mortality rate in the present study was 20.6%. In a prospective study by Mahmoud et al. on predictors of outcome in an Egyptian pediatric intensive care unit that included 451 patients, an ICU mortality rate of 37.9% was reported. This was significantly higher than the rate we observed [6]. A study from the University of Gondar Comprehensive Specialized in northwest Ethiopia examined mortality rates and predictors for children admitted to the pediatric intensive care unit and found a mortality rate of 32.6% [11]. A study in India also found a mortality rate of 23.24% [12]. However, a study in a resource-rich country found a much lower mortality rate of 6.54%, which may be due to differences in care, sample size, and study design [13].

Most (67.7%) of our admissions came from emergency department, followed by ward 49 (22.0%). The cumulative incidence of death was higher in patients transferred from the ward to the intensive care unit (34.7%). This is consistent with a study reported by Basheer et al. A study conducted in Pakistan found that emergency cases were the leading cause of ICU patients (267, 50.3%), followed by patients transferred to the ward (264, 49.7%) [14]. Furthermore, patients admitted from pediatric wards had a higher mortality rate compared to patients admitted from the emergency department (14.9 vs. 11.7%) [14].

The cumulative incidence of death in our study was higher in patients with cardiac diseases (40.0%), followed by oncologic diseases (35.0%) and infectious diseases (27.9%). Likewise, a study by Alkhalif et al. found a highest mortality in patients with immunological diseases (40.9%), followed by cardiovascular diseases (13%), and infection-related diseases (11.25%) [13]. In contrast to our results, other studies showed that respiratory diseases were the largest contributor to ICU mortality [7,14]. The difference may be that most of these patients had chronic lung disease secondary to bronchopulmonary dysplasia, cystic fibrosis, pulmonary tuberculosis, and interstitial diseases, the course of which was complicated by recurrent infections [7,14].

In our study, children with cardiac problems had 3.3 times higher mortality (AHR [95% CI] = 3.3 [1.3, 8.0], p = 0.010) than non-cardiac patients. This finding was not in keeping with an Egyptian study in which, the presence of kidney injury (AOR = 11.6, 95% CI (1.82–74.2), p = 0.009) and multiorgan dysfunction (AOR = 44.85, 95% CI (14.98–134.29), p < 0.001), a significantly increased risk of intensive care unit mortality was found [15]. The disparity in reported outcomes may be due to differences in disease severity and comorbidities.

Children with a reduced level of consciousness (GCS between 9 and 14 and GCS < 9) had 2.2- and 2.5-times higher mortality, respectively, than children with a normal level of consciousness (GCS of 15). These results were consistent with other studies conducted in Ethiopia, Iran, and Korea [7,16–19]. Low GCS is considered a strong predictor of mortality in intensive care patients. The possible explanation may not only be related to the severity of the disease. Instead, other factors may influence the prognosis, such as concomitant use of sedatives and an undetected hypoxic event.

The last, but not least important, predictor of time to death in the pediatric intensive care unit in this study was intubation with mechanical ventilation. Children with an endotracheal tube had a risk of death about five times higher than children without an endotracheal tube. The median survival time for children with an endotracheal tube was 9 days. Several studies in developing countries showed that the need for mechanical ventilation is a strong predictor of mortality in children [8,16,17,20]. However, a recent study evaluating survival status and predictors of mortality in selected tertiary care hospitals in Ethiopia shown contradictory results. They found that the use of mechanical ventilation was associated with reduced mortality (AHR: 0.45; 95% CI: 0.21, 0.92; P = 0.04). In addition, sedative use (AHR: 2.40; 95% CI: 1.16, 4.95; P = 0.02) was associated with an increased risk of ICU mortality [7]. The disease type, MV weaning process, natural history of the disease, and length of the ICU stay could explain the difference.

### Limitation of the study

A possible limitation of our study is its retrospective nature and the fact that it was conducted in a single center. Due to the retrospective nature of the study, we could not get adequate data related to sociodemographic characteristics of the parents, additional signs and symptoms of a disease and laboratory results.

## Conclusion

The mortality rate was high in our study. Cardiac patients were the most common cause of death in the intensive care unit, followed by oncological or infectious cases. The study results showed that cardiac patients, the presence of a low GCS score, and the need for intubation were significant and independent predictors of mortality. Providing due attention to all patients, especially those requiring high levels of care, can improve the outcome of patients admitted to the intensive care unit.

## Recommendation

Healthcare professionals should prioritize timely assessment of pediatric patients with cardiac problems, implement evidence-based interventions, monitor Glasgow Coma Scale (GCS) results, and explore alternative airway management strategies. Medical directors must develop guidelines, monitor outcomes, and provide ongoing training. Health departments should establish robust data collection systems, launch awareness campaigns, and allocate resources to pediatric intensive care units. Government agencies must play a critical role in developing and collaborating on policies to improve pediatric cardiac care.

## Supporting information

S1 File The PICU survival dataset in EXCEL file format. (XLSX)

## Abbreviations

ACSH: Ayder Comprehensive Specialized Hospital
AHR: Adjusted Hazard Ratio
AOR: Adjusted Odds Ratio
CCD: Chief Clinical Director
CHR: Crude Hazard Ratio
CI: Confidence Interval
CNS: Central Nervous System
GCS: Glasgow Coma Scale
HAI: Hospital Acquired Infection
HIC: High Income Country
ICU: Intensive Care Unit
IQR: Inter-Quartile Range
IRB: Institutional Review Board
LMICs: Low- and Middle-Income Countries
MV: Mechanical Ventilator
PICU: Pediatric Intensive Care Unit
VIF: Variance Inflation Factor
